# Orelabrutinib in combination with pomalidomide for the successful treatment of primary vitreoretinal lymphoma: a case report

**DOI:** 10.3389/fmed.2026.1806373

**Published:** 2026-08-07

**Authors:** Yanli Lai, Pengyao Lin, Lixia Sheng, Guifang Ouyang, Bo Li, Ningning Wu, Shasha You

**Affiliations:** 1 Department of Hematology, The First Affiliated Hospital of Ningbo University, Ningbo, Zhejiang, China; 2 Department of Ophthalmology, The First Affiliated Hospital of Ningbo University, Ningbo, Zhejiang, China; 3 Stem Cell Laboratory, Department of Hematology, The First Affiliated Hospital of Ningbo University, Ningbo, Zhejiang, China

**Keywords:** blood-ocular barrier, BTK inhibitors, methotrexate toxicity, orelabrutinib, pomalidomide, primary vitreoretinal lymphoma

## Abstract

**Background:**

Primary vitreoretinal lymphoma (PVRL) is a rare intraocular malignancy, often masquerading as chronic uveitis, which poses significant diagnostic and therapeutic challenges.

**Case presentation:**

A 55-year-old male with 1 year of progressive bilateral vision loss was initially misdiagnosed as having bilateral uveitis and received ineffective corticosteroid therapy at an outside medical center. Subsequent diagnostic vitrectomy of the right eye confirmed bilateral PVRL. He received intravitreal methotrexate (MTX) injections for treatment but developed MTX-induced toxic keratitis and progressive cataract, necessitating discontinuation of MTX. He was transitioned to systemic therapy with anti-CD20 monoclonal antibody (discontinued due to pulmonary infection) plus oral pomalidomide and orelabrutinib, which achieved complete clearance of vitreous disease, normalization of aqueous IL-10, and sustained clinical remission without significant hematologic or cardiac toxicities.

**Conclusion:**

Precise diagnosis and individualized treatment regimens are required for the therapy of PVRL. Besides, the orelabrutinib–pomalidomide combination represents a promising, well-tolerated targeted and chemo free therapy strategy for PVRL.

## Introduction

Primary vitreoretinal lymphoma (PVRL) is a rare, high-grade non-Hodgkin lymphoma, predominantly of the diffuse large B-cell type, and is considered a subset of primary central nervous system lymphoma (PCNSL) (1). It most commonly affects older adults and presents with progressive vision loss, vitreous opacities, and subretinal infiltrates (2). The clinical presentation is notoriously nonspecific, often leading to misdiagnosis as chronic uveitis or other intraocular inflammatory conditions, resulting in significant diagnostic delays and inappropriate initial treatments (3–5). Diagnosis relies on a high index of suspicion, multimodal imaging (notably optical coherence tomography), and molecular or cytopathological analysis of vitreous or aqueous humor samples, with MYD88 mutation serving as a valuable diagnostic marker and the interleukin-10 (IL-10)/IL-6 ratio contributing to a high index of suspicion (1, 6, 7).

Therapeutic management of PVRL is complex and not standardized (3). First-line local therapy often involves intravitreal injections of methotrexate (MTX) or rituximab. However, these regimens are protracted, require frequent invasive procedures, and are associated with notable ocular toxicity, including drug-induced keratitis, cataract acceleration, and elevated intraocular pressure. Furthermore, local therapy alone does not address the high risk of concurrent or subsequent central nervous system (CNS) involvement, which occurs in a majority of patients and dictates prognosis (8). Systemic strategies, particularly those capable of crossing the blood–brain and blood-retinal barriers, are therefore of paramount interest.

Bruton’s tyrosine kinase (BTK) is a crucial effector molecule in B-cell receptor signaling. PCNSL and PVRL frequently exhibit an activated B-cell-like phenotype with recurrent mutations in *MYD88* and *CD79B*, creating a strong biological rationale for BTK inhibitors (BTKis) therapy (9, 10). BTKis including ibrutinib have demonstrated high response rates in PCNSL, proving their ability to penetrate the blood–brain barrier (11, 12). Their efficacy and penetration into the ocular compartment, governed by the blood-retinal barrier, are less documented but theoretically promising.

Herein, we report a unique case of bilateral PVRL successfully treated with the novel BTKi orelabrutinib in combination with the immunomodulatory agent pomalidomide, following severe adverse events of intravitreal MTX. This case highlights the potential of this all-oral, targeted combination as an effective and well-tolerated therapeutic option.

## Case presentation

A 55-year-old male presented with a 1-year history of progressive bilateral vision loss. He had been diagnosed with bilateral uveitis at an outside facility in May 2021 and treated with corticosteroids without improvement. A diagnostic right eye vitrectomy confirmed a diagnosis of bilateral PVRL. He received three weekly intravitreal injections of MTX (400 μg) before being transferred to our institution.

**Figure 1:**
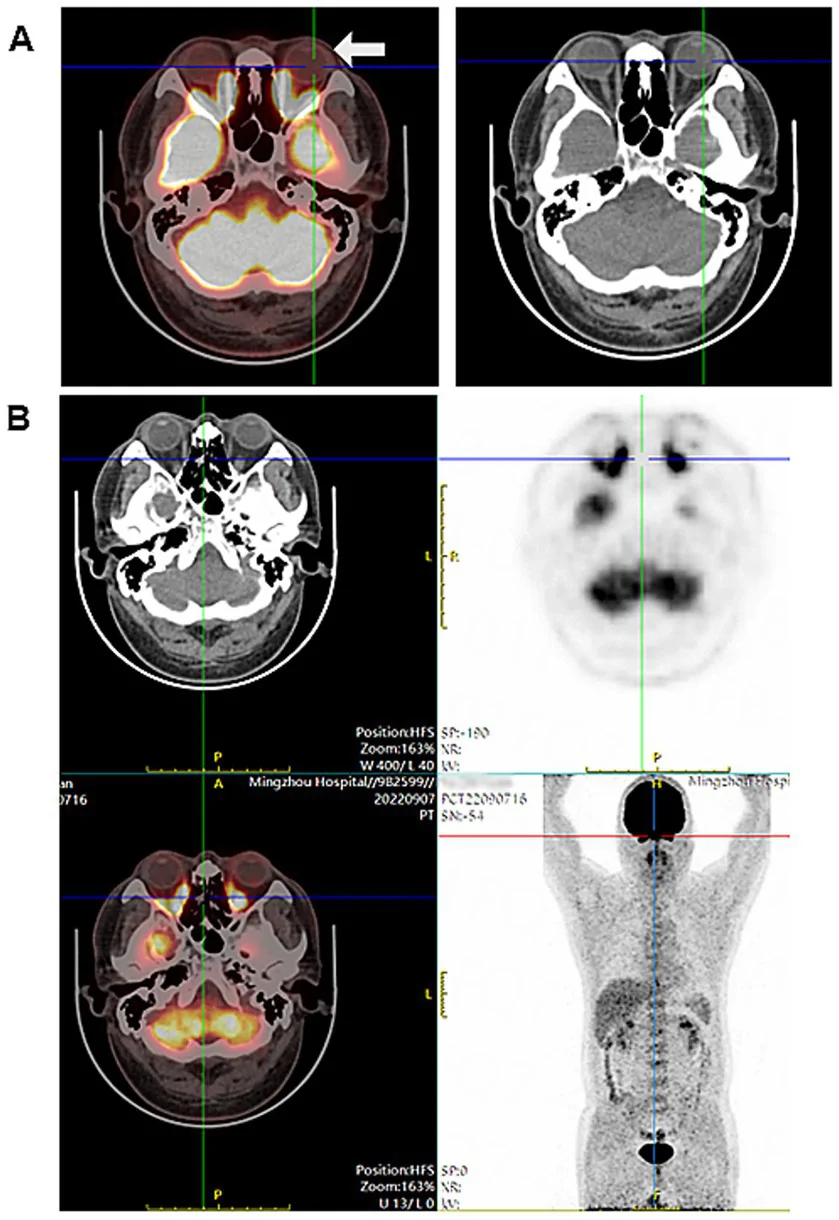
Fundoscopy, B-scan ultrasonography and optical coherence tomography (OCT) examinations during treatment. **(A)** Results from admission on September 9th, 2022. Fundus photography revealed a retinal infiltrate in the temporal side of the right eye, and the left fundus was blurred due to severe vitreous opacity. B-scan ultrasonography shows mild vitreous opacity in the right eye and severe vitreous opacity in the left eye. OCT shows enlarged nodular hyperreflective lesions that can be observed in the RPE layer and above the RPE, and in the infrared image of OCT shows visible mottled, granular “leopard spot” changes. **(B)** Results after the eleventh intravitreal MTX injection on October 14, 2022. The retinal infiltrate was reduced, the vitreous opacity was relieved, and the structure of the outer retinal layer was partially recovered.

**Figure 2:**
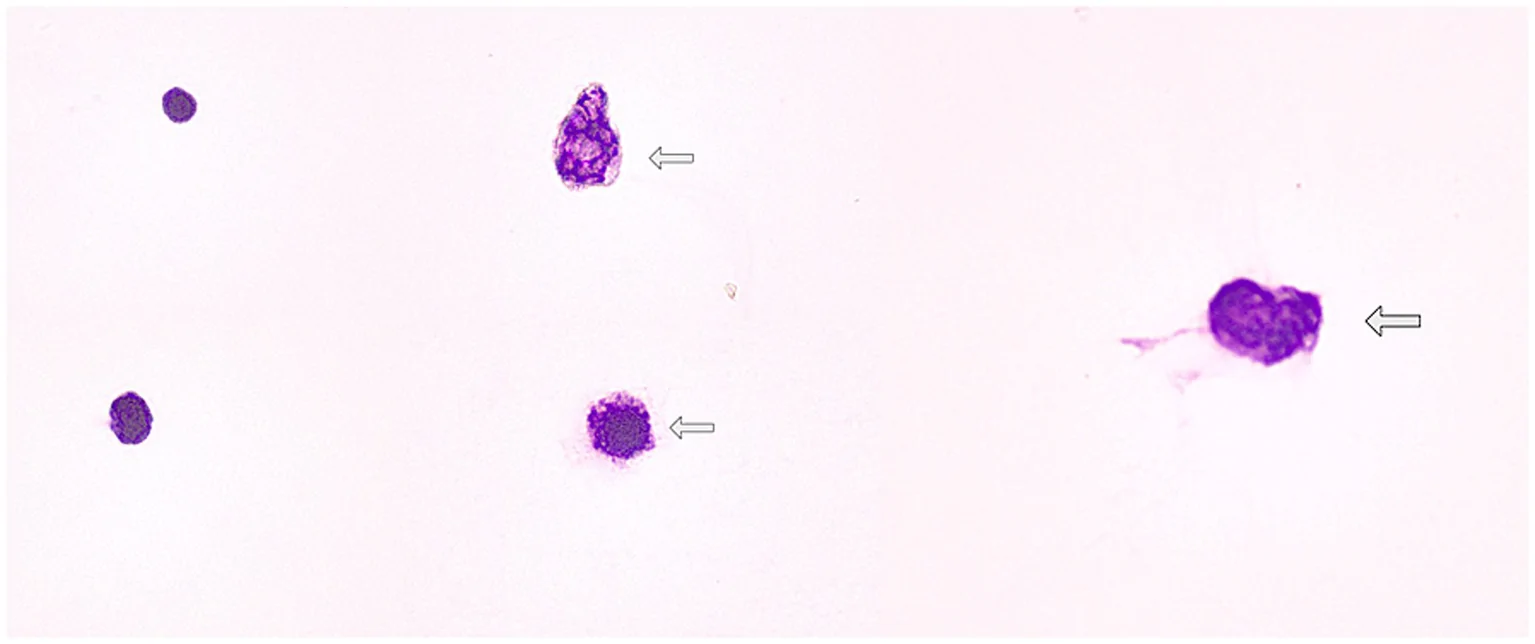
Cytology of the vitreous fluid. A dense infiltrate of atypical lymphoid cells with blastic features. Note the large cell size, moderate to abundant basophilic cytoplasm with occasional projections, irregular nuclei with open chromatin, and prominent nucleoli (1–3 per cell). Findings are consistent with lymphoma. ×100 magnification.

**Figure 3:**
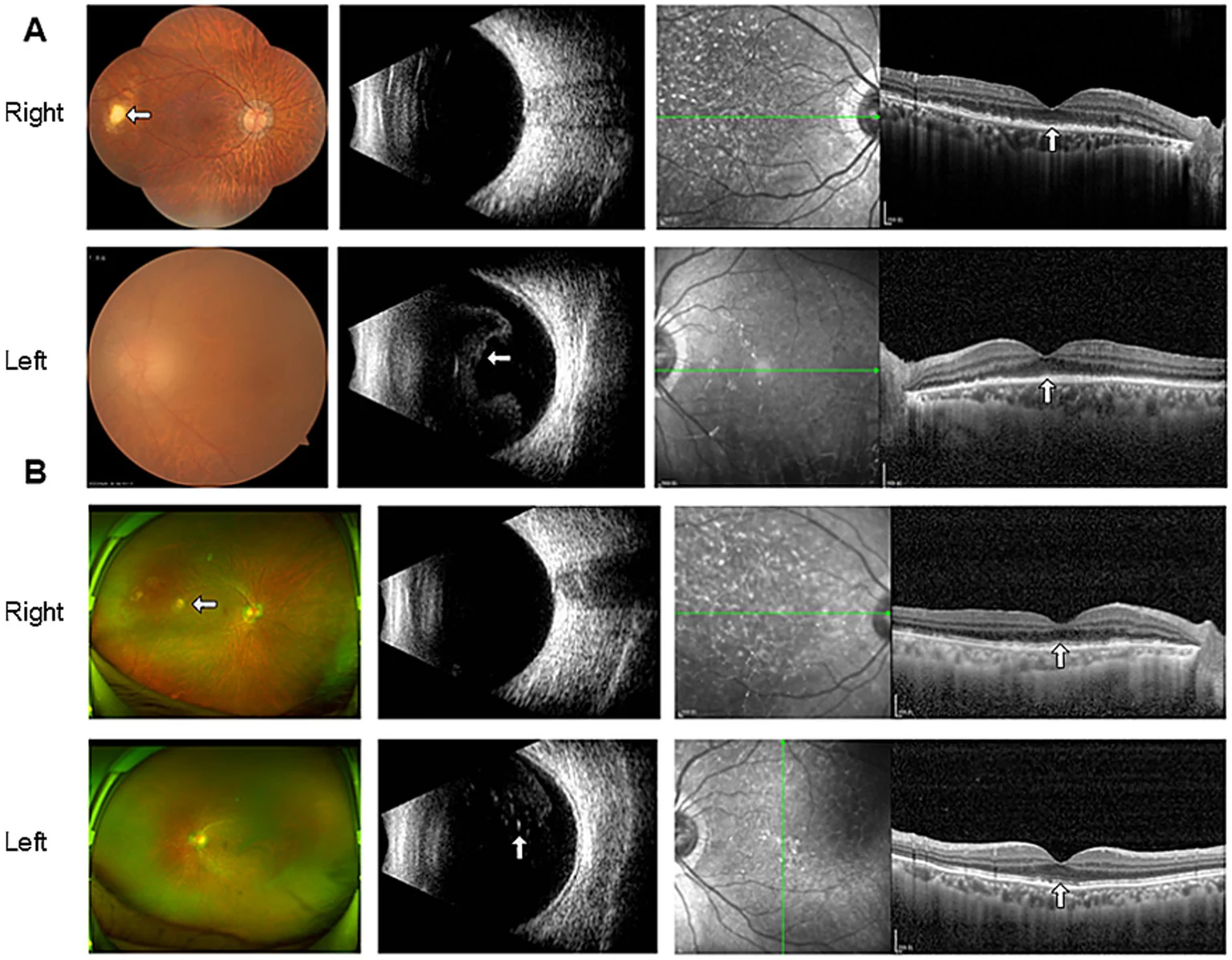
Positron emission tomography-computed tomography (PET/CT) results. The density distribution of the vitreous humor in both eyes is uneven, with a slight increase in FDG uptake, and SUVmax/av. = 2.4/1.8. No abnormal FDG uptake was observed in other systemic organs, confirming that the disease was confined to the ocular compartments. **(A)** Results from admission on September 9th, 2022. **(B)** Results after the eleventh injection on October 14, 2022.

On admission, his best-corrected visual acuity (BCVA) was 20/80 in both eyes. Slit-lamp examination revealed conjunctival hyperemia, posterior pigmentary keratic precipitates, and lens opacities. Aqueous humor sampling via 30-gauge anterior chamber paracentesis revealed markedly elevated IL-10 concentrations of 1,946.4 pg/mL (right eye) and 325.8 pg/mL (left eye; normal reference range: 0–5.0 pg/mL). The corresponding IL-10/IL-6 ratios were 19.2 in the right eye and 16.6 in the left eye. Fundoscopy revealed a retinal infiltrate in the temporal side of the right eye, and the left fundus was blurred due to severe vitreous opacity. B-scan ultrasonography showed mild vitreous opacity in the right eye and severe vitreous opacity in the left eye. OCT displayed enlarged nodular hyperreflective lesions that can be observed in the retinal pigment epithelium (RPE) layer and above the RPE, and in the infrared image of OCT shows visible mottled, granular “leopard spot” changes. (Figure 1A). Given the invasive nature of the procedure and the patient’s history of severe ocular toxicity following prior intravitreal injections, a repeat diagnostic vitrectomy was not performed. Instead, a vitreous aspiration was carried out for re-evaluation to confirm the disease status. Cytology of the vitreous fluid showed a dense infiltrate of atypical lymphoid cells with blastic features. The large cell size, moderate to abundant basophilic cytoplasm with occasional projections, irregular nuclei with fine chromatin, and prominent nucleoli (1–3 per cell) indicated that they were consistent with lymphomatous infiltration (Figure 2). The *MYD88* L265P mutation test result was negative. Positron Emission Tomography-Computed Tomography (PET-CT), confirmed the disease was confined to the eyes, leading to a diagnosis of primary vitreoretinal lymphoma with bilateral age-related cataracts (Figure 3). However, since brain MRI is more sensitive than PET-CT for detecting central nervous system (CNS) involvement, brain MRI examinations performed at an outside hospital 3 months prior to admission had already preliminarily ruled out CNS involvement.

He was initiated on a standard induction regimen of weekly intravitreal MTX (400 μg). After the eleventh injection on October 14, 2022, he developed significant drug-induced toxic keratitis and worsening cataracts (Figure 1B). By his twelfth treatment on October 21, 2022, his BCVA had deteriorated to 20/200 (right eye) prompting permanent discontinuation of MTX. Therapy was then transitioned to a systemic regimen (23 October 2022–27 November 2022) which consisted of intravenous dose of rituximab (700 mg, iv, once a month), oral pomalidomide (4 mg daily) and orelabrutinib (150 mg daily). Rituximab was temporarily held due to an episode of pneumonia to mitigate infection risk. The patient has since been maintained on the oral therapy of orelabrutinib (150 mg daily) and pomalidomide (4 mg daily).

**Figure 4:**
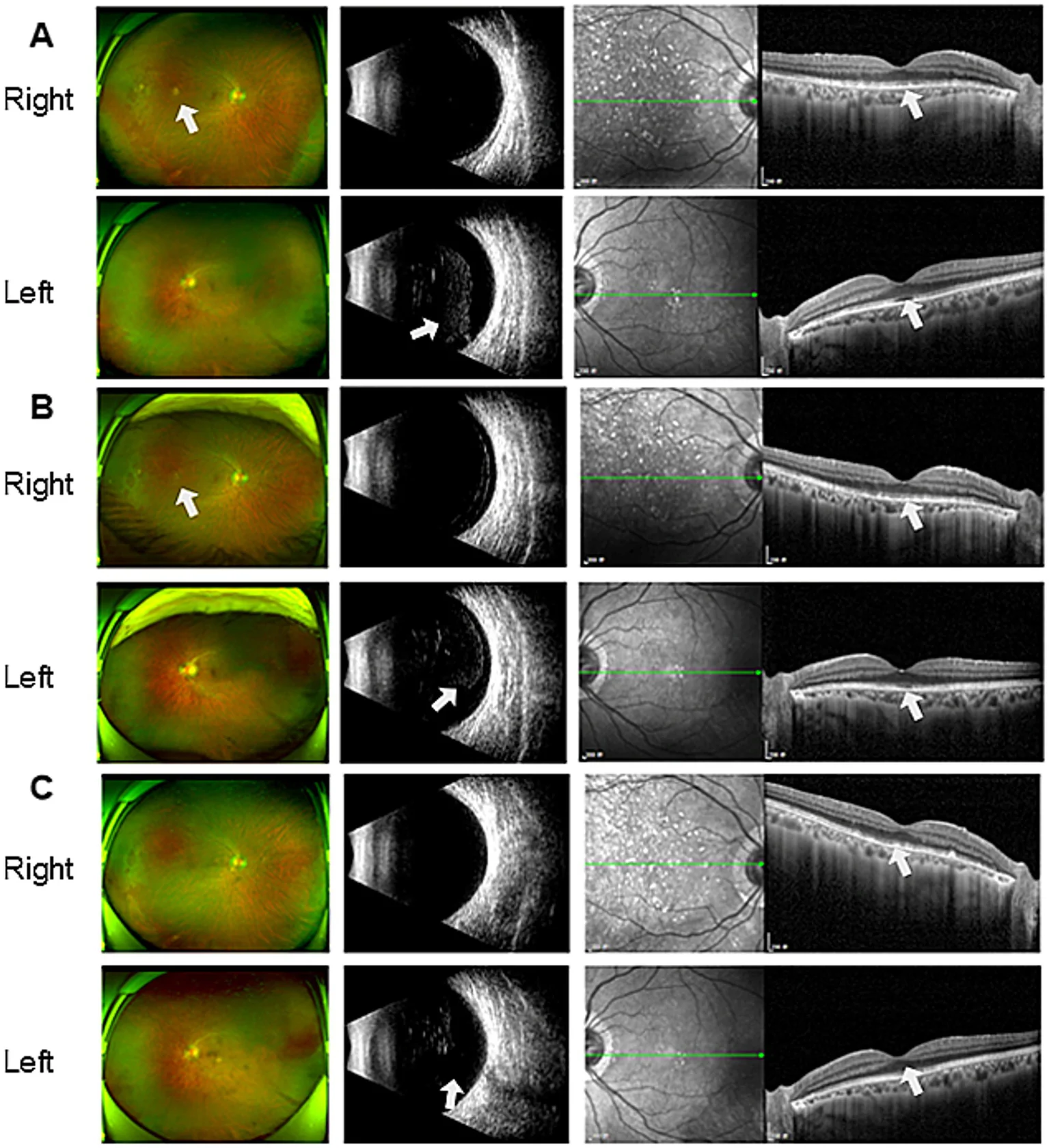
Fundoscopy, B-scan ultrasonography and optical coherence tomography (OCT) examinations during follow-up. **(A)** Results after oral medications treatment for 1 month on November 28th, 2022, the retinal infiltrate was significantly reduced compared to the onset of the disease, the vitreous opacity was relieved, and the structure of the outer layer of the retina has recovered better than before. **(B)** Results after oral medications treatment for 2 months on January 4th, 2023. The retinal infiltrate gradually disappeared, the vitreous opacity was relieved, the structure of the outer layer of the retina recovered. **(C)** Results after oral medications treatment for 6 months on May 10th, 2023, the retinal infiltrate completely disappeared, and the vitreous opacity was significantly relieved, the structure of the outer layer of the retina has mostly recovered, especially in the left eyes.

At a two-month follow-up, a marked reduction in vitreous opacities and subretinal/RPE lesions, with partial restoration of the outer retinal architecture was observed (Figures 4A,B). The aqueous humor IL-10 level in the right eye normalized to 4.6 pg/mL, and the IL-10/IL-6 ratio was <1 in both eyes, indicating biochemical response. After 6 months of combination therapy, the patient achieved complete clinical and radiological remission (Figure 4C), showing improved visual acuity. Although the patient’s visual acuity improved (20/250 OD, 20/40 OS), the ultimate visual outcome was suboptimal due to structural retinal damage (such as subretinal exudation and subsequent RPE atrophy), which underscores the critical importance of early intervention in preventing irreversible vision loss in PVRL. Notably, he experienced no grade ≥2 adverse events typically associated with BTK is or immunomodulatory drugs, such as bleeding, atrial fibrillation, diarrhea, or myelosuppression.

## Discussion

This case underscores two pervasive challenges in PVRL management: its diagnostic elusiveness and the toxicity of conventional local chemotherapy. Our patient’s initial misdiagnosis as uveitis is a classic presentation pitfall, extensively documented in the literature. PVRL is a great masquerader, and a high index of suspicion is required in cases of atypical, steroid-refractory “uveitis,” particularly in older patients. Diagnostic delays can lead to sight-threatening complications and increase the risk of CNS dissemination (3, 13, 14). Besides, although the patient’s visual acuity improved, the final visual outcome was suboptimal due to structural retinal damage, such as subretinal infiltrates and subsequent RPE atrophy, highlighting that early intervention is crucial to prevent irreversible visual loss in PVRL.

The second challenge is illustrated by the patient’s adverse reaction to intravitreal MTX, the most commonly employed local chemotherapeutic agent. While effective, the protracted course of frequent injections often leads to cumulative toxicity (8, 15, 16). As seen in our case and supported by other reports, MTX can induce severe toxic keratopathy, accelerate cataract formation, and cause significant patient discomfort, sometimes necessitating treatment cessation (3, 17). The toxic keratitis observed in our patient could be partially attributed to the cumulative toxicity of MTX, as well as potential MTX reflux onto the anterior corneal surface, which has been reported to contribute to corneal toxicity (18). Anterior chamber tap after injection may help mitigate this risk. These limitations highlight the need for alternative, less toxic yet effective therapies.

Regarding the systemic treatment strategy, although combined systemic chemotherapy has been suggested to prevent CNS progression in PVRL (19), we did not consider this regimen as a first-line option for this patient due to the high risk of infection associated with chemotherapy, as well as the initial priority of controlling intraocular inflammation with local therapy. However, for PVRL patients without CNS involvement, systemic treatment plays a crucial role in potentially eradicating occult disease and preventing CNS dissemination, which warrants further investigation.

The transition to an all-oral regimen of orelabrutinib and pomalidomide in our patient proved to be both effective and well-tolerated. This success is grounded in a strong biological rationale. Orelabrutinib is a highly selective, next-generation BTK inhibitor (20). Its small molecular size and properties may facilitate penetration of the blood-retinal barrier, a critical requirement for treating intraocular disease. This is analogous to the proven CNS penetrance of BTK is in PCNSL. By inhibiting BTK, a key node in the B-cell receptor signaling pathway frequently hyperactive in PVRL due to mutations like *MYD88* L265P, orelabrutinib directly targets the lymphoma’s proliferative machinery (21, 22). The synergy between targeted inhibition (orelabrutinib) and immunomodulation (pomalidomide) may explain the rapid and durable response observed, achieving both vitreous clearance and biochemical normalization (23). Additionally, prior serial intravitreal MTX injections and one course of intravenous rituximab may have jointly debulked intraocular lesions and laid the foundation for subsequent durable response.

The oral dual-agent regimen exhibited favorable tolerability in this single patient, with no atrial fibrillation, bleeding or diarrhea frequently linked to off-target effects of first-generation ibrutinib, and oral administration also improves quality of life by avoiding repeated invasive intravitreal injections (24–26). While a prior case from Li et al. (27) reported comparable efficacy of BTKi-containing therapy in a similarly middle-aged PVRL patient, clinical evidence supporting this chemo-free strategy remains extremely limited. As this study only represents an isolated case, we cannot draw generalizable conclusions regarding its long-term efficacy and CNS protective capacity. Large prospective cohorts are necessary to further validate its clinical value.

## Conclusion

In summary, PVRL remains a diagnostic and therapeutic dilemma. This case report highlights that intolerance or toxicity from first-line intravitreal chemotherapy should not lead to a therapeutic dead-end. The combination of orelabrutinib and pomalidomide represents a novel, effective, and well-tolerated all-oral systemic strategy for PVRL. However, this report is a single-patient observation. Larger prospective studies are warranted to confirm these findings.

## Data Availability

The original contributions presented in the study are included in the article/Supplementary material, further inquiries can be directed to the corresponding author.

## References

[1] Chan CC Rubenstein JL Coupland SE Davis JL Harbour JW Johnston PB . Primary vitreoretinal lymphoma: a report from an international primary central nervous system lymphoma collaborative group symposium. Oncologist. (2011) 16:1589–99. doi: 10.1634/theoncologist.2011-0210, 22045784 PMC3233294

[2] Carbonell D Mahajan S Chee SP Sobolewska B Agrawal R Bülow T . Consensus recommendations for the diagnosis of vitreoretinal lymphoma. Ocul Immunol Inflamm. (2021) 29:507–20. doi: 10.1080/09273948.2021.1878233, 34009095

[3] Zhao XY Cheng TT Meng LH Zhang WF Chen YX. Clinical features, diagnosis, management and prognosis of primary intraocular lymphoma. Front Oncol. (2022) 12:808511. doi: 10.3389/fonc.2022.808511, 35186744 PMC8851327

[4] He LF Zhang JD Chen XX Wei RL. Correction: Epidemiology and survival outcomes of patients with primary intraocular lymphoma: a population-based analysis. BMC Ophthalmol. (2023) 23:56. doi: 10.1186/s12886-023-02781-z, 36759809 PMC9909847

[5] Kreher S Strehlow F Martus P Roth P Hertenstein B Röth A . Prognostic impact of intraocular involvement in primary CNS lymphoma: experience from the G-PCNSL-SG1 trial. Ann Hematol. (2015) 94:409–14. doi: 10.1007/s00277-014-2212-z, 25217230

[6] Huang RS Mihalache A Popovic MM Cruz-Pimentel M Pandya BU Muni RH . Diagnostic methods for primary vitreoretinal lymphoma: a systematic review. Surv Ophthalmol. (2024) 69:456–64. doi: 10.1016/j.survophthal.2023.12.001, 38163550

[7] Chen K Li X Wang D Ma Y Chen B Wang Q . The diagnostic value of IL-10 and IL-6 level in vitreous fluid and aqueous humor for vitreoretinal lymphoma. Clin Chim Acta. (2021) 515:21–6. doi: 10.1016/j.cca.2020.12.035, 33387464

[8] Gu J Jiang T Liu S Chen X Wang Z Zhang P . Longitudinal analysis of ocular manifestation and interleukin during intravitreal treatment of vitreoretinal lymphoma with methotrexate. Am J Ophthalmol. (2023) 251:189–96. doi: 10.1016/j.ajo.2023.03.010, 36963600

[9] Bonzheim I Giese S Deuter C Süsskind D Zierhut M Waizel M . High frequency of MYD88 mutations in vitreoretinal B-cell lymphoma: a valuable tool to improve diagnostic yield of vitreous aspirates. Blood. (2015) 126:76–9. doi: 10.1182/blood-2015-01-620518, 25900979

[10] Hiemcke-Jiwa LS Ten Dam-van Loon NH Leguit RJ Nierkens S Ossewaarde-van Norel J de Boer JH . Potential diagnosis of vitreoretinal lymphoma by detection of MYD88 mutation in aqueous humor with ultrasensitive droplet digital polymerase chain reaction. JAMA Ophthalmol. (2018) 136:1098–104. doi: 10.1001/jamaophthalmol.2018.2887, 30027272 PMC6233833

[11] Alcantara M Chevrier M Jardin F Schmitt A Houillier C Oberic L . Phase IB part of LOC-R01, a LOC network non-comparative randomized phase IB/II study testing R-MPV in combination with escalating doses of lenalidomide or ibrutinib for newly diagnosed primary central nervous system lymphoma (PCNSL) patients. J Hematol Oncol. (2024) 17:86. doi: 10.1186/s13045-024-01606-w, 39300447 PMC11414093

[12] Alcantara M Chevrier M Jardin F Schmitt A Houillier C Oberic L . Correction: phase IB part of LOC-R01, a LOC network non-comparative randomized phase IB/II study testing R-MPV in combination with escalating doses of lenalidomide or ibrutinib for newly diagnosed primary central nervous system lymphoma (PCNSL) patients. J Hematol Oncol. (2024) 17:98. doi: 10.1186/s13045-024-01620-y, 39427203 PMC11490990

[13] Takase H Arai A Iwasaki Y Imai A Nagao T Kawagishi M . Challenges in the diagnosis and management of vitreoretinal lymphoma—clinical and basic approaches. Prog Retin Eye Res. (2022) 90:101053. doi: 10.1016/j.preteyeres.2022.101053, 35210172

[14] Soussain C Malaise D Cassoux N. Primary vitreoretinal lymphoma: a diagnostic and management challenge. Blood. (2021) 138:1519–34. doi: 10.1182/blood.2020008235, 34036310

[15] Zhou X Zhou X Shi H Lai J Wang Q Li Y . Reduced frequency of intravitreal methotrexate injection lowers the risk of keratopathy in vitreoretinal lymphoma patients. BMC Ophthalmol. (2020) 20:189. doi: 10.1186/s12886-020-01464-3, 32397978 PMC7216350

[16] Zhao H Wang X Mao Y Peng X. Longitudinal observation of OCT imaging is a valuable tool to monitor primary vitreoretinal lymphoma treated with intravitreal injections of methotrexate. BMC Ophthalmol. (2020) 20:10. doi: 10.1186/s12886-019-1300-1, 31906917 PMC6945433

[17] Frenkel S Hendler K Siegal T Shalom E Pe'er J. Intravitreal methotrexate for treating vitreoretinal lymphoma: 10 years of experience. Br J Ophthalmol. (2008) 92:383–8. doi: 10.1136/bjo.2007.127928, 18303160

[18] Pederzolli M Giglio F Cicinelli MV Marchese A Modorati G Mastaglio S . Case report: intravitreal methotrexate in intraocular acute lymphoblastic leukemia. Front Oncol. (2022) 12:951362. doi: 10.3389/fonc.2022.951362, 36106118 PMC9464940

[19] Akiyama H Takase H Kubo F Miki T Yamamoto M Tomita M . High-dose methotrexate following intravitreal methotrexate administration in preventing central nervous system involvement of primary intraocular lymphoma. Cancer Sci. (2016) 107:1458–64. doi: 10.1111/cas.13012, 27412324 PMC5084671

[20] Alu A Lei H Han X Wei Y Wei X. BTK inhibitors in the treatment of hematological malignancies and inflammatory diseases: mechanisms and clinical studies. J Hematol Oncol. (2022) 15:138. doi: 10.1186/s13045-022-01353-w, 36183125 PMC9526392

[21] Cao XX Jin J Fu CC Yi SH Zhao WL Sun ZM . Evaluation of orelabrutinib monotherapy in patients with relapsed or refractory Waldenström's macroglobulinemia in a single-arm, multicenter, open-label, phase 2 study. EClinicalMedicine. (2022) 52:101682. doi: 10.1016/j.eclinm.2022.101682, 36313145 PMC9596308

[22] Deng T Zhang S Xiao M Gu J Huang L Zhou X. A single-centre, real-world study of BTK inhibitors for the initial treatment of MYD88(Mut) /CD79B(Mut) diffuse large B-cell lymphoma. Cancer Med. (2024) 13:e7005. doi: 10.1002/cam4.7005, 38457222 PMC10923040

[23] Richardson PG Perrot A San-Miguel J Beksac M Spicka I Leleu X . Isatuximab plus pomalidomide and low-dose dexamethasone versus pomalidomide and low-dose dexamethasone in patients with relapsed and refractory multiple myeloma (ICARIA-MM): follow-up analysis of a randomised, phase 3 study. Lancet Oncol. (2022) 23:416–27. doi: 10.1016/S1470-2045(22)00019-5, 35151415

[24] Kater AP Kersting S Dubois JM van der Holt B da Cunha-Bang C Te Raa D . Fixed-duration ibrutinib-venetoclax with MRD-guided ibrutinib-obinutuzumab intensification in first-line chronic lymphocytic leukaemia (HOVON 158/NEXT STEP): primary analysis of a multicentre, open-label, phase 2 trial. Lancet Haematol. (2025) 12:e935–45. doi: 10.1016/S2352-3026(25)00288-1, 41338862

[25] Khouja M Genuardi E Ferrero S Laqua A Alessandria B Verhagen O . Noninvasive genotyping and early disease dynamics demonstrate the efficacy of ibrutinib in combination with immunochemotherapy in patients with mantle cell lymphoma treated in the TRIANGLE trial. Leukemia. (2026) 40:95–105. doi: 10.1038/s41375-025-02787-0, 41184633 PMC12789019

[26] Ghia P Ysebaert L Janssens A Stilgenbauer S Fouad M Schioppa CA . Overall survival outcomes with first-line continuous ibrutinib and fixed-duration ibrutinib-venetoclax treatments in patients with chronic lymphocytic leukemia: comparison with an age-matched European population. Hema. (2025) 9:e70246. doi: 10.1002/hem3.70246, 41164354 PMC12560247

[27] Li Y Qiao R Zhang X Li Y. Orelabrutinib and rituximab regimen combined with intravitreal methotrexate for treatment of primary vitreoretinal lymphoma: a case report and literature review. Front Oncol. (2026) 16:1800529. doi: 10.3389/fonc.2026.1800529, 42003971 PMC13082968

